# Supplementary material for: Hammerhead-type FXR agonists induce an eRNA FincoR that ameliorates nonalcoholic steatohepatitis in mice
Source: bioRxiv. 2024 Feb 8:2023.11.20.567833. Originally published 2023 Nov 21. Preprint. [Version 2] doi: 10.1101/2023.11.20.567833 (PMC10690184; doi:10.1101/2023.11.20.567833)

bioRxiv preprint doi: https://doi.org/10.1101/2023.11.20.567833; this version posted February 8, 2024. The copyright holder for this preprint (which was not certified by peer review) is the author/funder, who has granted bioRxiv a license to display the preprint in perpetuity. It is made available under aCC-BY 4.0 International license.

Supplementary Figure Legends

Figure 1- figure supplement 1. (A, B) Examples of FXR-regulated eRNAs produced near the genes *Hes1* (A) and *Slc35g1* (B) were shown. (C) Time course expression of *FincoR*. C57BL/6 male mice were fasted overnight and injected *i.p.* with vehicle or GW4064 (30 mg/kg) for 1, 3, 6,12 h. Livers were collected at the indicated time points (n=3/group) and *FincoR* and mRNA levels of *Shp* and *Gcnt1* were measured. Data are presented as mean ± SEM. Statistical significance was determined by the two-way ANOVA Sidak's multiple comparisons test with **p* < 0.05 and ****p* < 0.001.

Figure 1- figure supplement 2. (A) Volcano plot showing the differential expressed genes (DEGs) after GW4064 treatment (DESeq2 FDR<0.05). The numbers refer to the number of genes up- or down-regulated. (B) Bar plot showing the enriched Gene Ontology in terms of biological processes for up-regulated genes.

Figure 2- figure supplement 1. (A) FXR protein levels in the livers isolated from FXR-Flox and FXR-LKO mice are shown. (B) Validation of hepatic FXR-dependent induction of *FincoR* by qPCR (n = 3 mice). The *Shp* gene was used as a control. Data are presented as mean ± SEM. Statistical significance was determined by the two-way ANOVA Tukey's multiple comparisons test with **p* < 0.05 and ***p* < 0.01. (C) Metagene plots showing H3K27ac, H3K4me1, FXR, and RXR**α** ChIP-Seq profiles centered on up-regulated eRNAs.

Figure 5- figure supplement 1. (A) Left: experimental scheme for the *FincoR* loss of function experiments. Right: illustration demonstrating the sequence targeted by the sgRNA in relation to the transcriptional and epigenetic profile. (B) The genomic DNAs from liver, spleen, intestine, brain, heart, muscle, kidney, lung and adipose tissue were

bioRxiv preprint doi: https://doi.org/10.1101/2023.11.20.567833; this version posted February 8, 2024. The copyright holder for this preprint (which was not certified by peer review) is the author/funder, who has granted bioRxiv a license to display the preprint in perpetuity. It is made available under aCC-BY 4.0 International license.

isolated and PCR was performed using the primers (**Supplementary Table S6**) to verify tissue-specific knock-out. (C-G) RNA-seq profiles of expression of hepatic *Prune2* (C)*,* *PPP1r3g* (D)*, Igfbp2* (E)*, Eda2r* (F) *and Fndc1* (G) are shown (n=2/group).

Figure 7- figure supplement 1. The effects of *FincoR* downregulation on NASH pathologies. Male Cas9 mice were fed with a NASH diet for 12 weeks. Then these mice were randomly assigned to 2 groups and infected with adenovirus expressing sgRNA for *FincoR* or control. The tissues were collected 2 weeks later. Liver histology analysis was performed and representative images were shown. Scale bar (50 μm).

Supplementary Figure S1. Hepatic expression of *FincoR* is elevated in liver disease associated with inflammation and fibrosis. (A) C57BL/6 mice were fed with a high fat diet for 12 weeks. The liver RNAs were extracted and *FincoR* expression was measured (n=5/group). (B) C57BL/6 mice were fed with a high fat diet with high fructose water for 12 weeks. The liver RNAs were extracted and *FincoR* expression was measured (n=7/group). (C) C57BL/6 mice were treated with ANIT (75 mg/kg) for 48 h and then sacrificed after 5 h of fasting. The liver RNAs were extracted, and *FincoR* expression was measured (n=4~5/group). (D) C57BL/6 mice were bile duct ligated for 1 day or 3 days. They were then sacrificed after 5 h of fasting. The liver RNAs were extracted and *FincoR* expression was measured (n=4~5/group). (E) *FincoR* conservation between mice and human as displayed in the UCSC Genome Browser. Red arrows indicate the conserved region. (F) Human lncRNA XR_007061585.1 with sequence similarity to mouse *FincoR* annotated in the NCBI genome data viewer. (G) Expression of lncRNA XR_007061585.1 in liver samples from normal individuals or patients with primary biliary cholangitis (PBC)

bioRxiv preprint doi: https://doi.org/10.1101/2023.11.20.567833; this version posted February 8, 2024. The copyright holder for this preprint (which was not certified by peer review) is the author/funder, who has granted bioRxiv a license to display the preprint in perpetuity. It is made available under aCC-BY 4.0 International license.

(n=14~15/group). (H) Expression of lncRNA XR_007061585.1 in liver samples from normal individuals or patients with NAFLD-associated steatosis (n=12~15/group). (A-D, G-H) Data are presented as mean ± SEM. Statistical significance was determined by the Student's t test with *$p < 0.05$, **$p < 0.01$ and ***$p < 0.001$.

Supplementary Figure S2. Hepatic genome browser tracks of FXR, RXRα, LXR, PPARα, and HNF4α binding peaks at the *FincoR* locus.

Supplementary Figure S3. PPARα occupancy in the *FincoR* enhancer region. C57BL/6 male mice were fasted overnight with or without refeeding for 3 h and then sacrificed. ChIP assays were performed in liver samples to detect PPAR**α** occupancy at the FXR binding peak region close to the transcription start site of *FincoR*.

bioRxiv preprint doi: https://doi.org/10.1101/2023.11.20.567833; this version posted February 8, 2024. The copyright holder for this preprint (which was not certified by peer review) is the author/funder, who has granted bioRxiv a license to display the preprint in perpetuity. It is made available under aCC-BY 4.0 International license.

Supplemental Tables

Supplemental Table S1. Sequencing data generated in this study

Name	Total reads	Uniquely mapped reads
WT Liver Veh RNA-Seq rep1	50887391	38072659
WT Liver GW RNA-Seq rep1	54020150	40595856
WT Liver Veh RNA-Seq rep2	51268164	38898913
WT Liver GW RNA-Seq rep2	51899892	39487241
Flox Liver Veh GRO-Seq	62960996	30305910
Flox Liver GW GRO-Seq	59210073	26116795
FXR KO Liver Veh GRO-Seq	34153537	14379417
FXR KO Liver GW GRO-Seq	36430101	15008281
WT Liver RNA-Seq rep1	96262381	81801494
WT Liver RNA-Seq rep2	104979915	88365481
FincoR KD Liver RNA-Seq rep1	91654076	75346167
FincoR KD Liver RNA-Seq rep2	90211420	74745807

Supplemental Table S2 is attached as an Excel file.

Supplemental Table S3. Public ChIP-Seq dataset

target	accession ID
FXR	https://genome.ucsc.edu/cgi-bin/hgCustom?hgsid=1577671033_RPWVA7jbx3rDswKOeo2dazTsXxe0&hgct_table=ct_FxrLiver_2971
H3K27ac	ENCFF001KMI
H3K4me1	ENCFF001KNF
LXR	GSM864669
RXRα	GSM864674
PPARα	GSM864671
HNF4α	GSM2055887

Supplemental Table S4 is attached as an Excel file.

Supplemental Table S5: Predicted RNA binding proteins (RBPs) binding to *FincoR*

RBP	Length	Motif	Domain	number of binding sites
KHDRBS1	7	UAAAAAG	KH	1
RBM38	7	UUGUGUG, GUGUGUG	RRM	3
YBX2	7	CACACCA	CSD	11
YBX3	7	CACACCA	CSD	11
PCBP1	6	CUUUCC	KH	1
KHSRP	6	UGCAUG	KH	2
PTBP1	6	CUCUCU	RRM	1
PTBP2	6	CUCUCU	RRM	1
4KZD	5	GAAAC	N/A	1
4KZE	5	GAAAC	N/A	1
4Q9Q	5	GAAAC	N/A	1
DAZL	5	GUUCU	RRM	6

bioRxiv preprint doi: https://doi.org/10.1101/2023.11.20.567833; this version posted February 8, 2024. The copyright holder for this preprint (which was not certified by peer review) is the author/funder, who has granted bioRxiv a license to display the preprint in perpetuity. It is made available under aCC-BY 4.0 International license.

MSI1	5	GUAGU	RRM	1
TLR3	5	AAAGG	LRR;TIR	12

Supplemental Table S6. Primer sequences used in this study

A. List of primer sequences for mouse qRT-PCR

**Gene**	**Forward Primer (5'-3')**	**Reverse Primer (5'-3')**
FincoR	GCAAAGCACCTTCTAGCACA	GTCAGGGAGCTAACGAATGC
Gcnt1	GCATCGCATCCTGCTTTGATA	GGTCTGCCTTAACCCGACTC
Shp	TCTGCAGGTCGTCCGACTAT	CAGGCAGTGGCTGTGAGAT
Cyp7a1	GGGATTGCTGTGGTAGTGAGC	GGTATGGAATCAACCCGTTGTC
Cyp8b1	CCTCTGGACAAGGGTTTTGTG	GCACCGTGAAGACATCCCC
Srebp1c	GCAGCCACCATCTAGCCTG	CAGCAGTGAGTCTGCCTTGAT
Lpin1	CATGCTTCGGAAAGTCCTTCA	GGTTATTCTTTGGCGTCAACCT
Scd1	TTCTTGCGATACACTCTGGTGC	CGGGATTGAATGTTCTTGTCGT
Ifng	GCG TCA TTG AAT CAC ACC TG	GAC CTG TGG GTT GTT GAC CT
Ccl3	TTCTCTGTACCATGACACTCTGC	CGTGGAATCTTCCGGCTGTAG
Ccl2	TTAAAAACCTGGATCGGAACCAA	GCATTAGCTTCAGATTTACGGGT
Ccr2	AAGAGGGCATTGGATTCACCACA	GCCGTGGATGAACTGAGGTAACA
Lcn2	GCAGGTGGTACGTTGTGGG	CTCTTGTAGCTCATAGATGGTGC
Col1a1	ATCGGTCATGCTCTCTCCAAACCA	ACTGCAACATGGAGACAGGTCAGA
Col1a2	CCTTTGTCAGAATACTGAGCAGC	GTAACTTCGTGCCTAGCAACA
Acta2	TCGGATACTTCAGCGTCAGGA	GTCCCAGACATCAGGGAGTAA
Eda2r	CACACTGCATAGTCTGCCCTC	GCCTTCTGGACCCGATTGA
Fndc1	GGGAGACATGGCAAACCTGT	TGGTAGGAGAGTATGTGGTGG
Ctsb	TCCTTGATCCTTCTTTCTTGCC	ACAGTGCCACACAGCTTCTTC
Ctss	GAAGTACGGCGTCTCATCTGG	CATGCCCACTTGGTAGGTATG
Bcl2	GTCGCTACCGTCGTGACTTC	CAGACATGCACCTACCCAGC
36b4	CCCTGAAGTGCTCGACATCA	TGCGGACACCCTCCAGAA
XR_007061585.1	TTGTCATCAAGCCCTGTTCA	TCTGCTTTGTCTGAGGACCA

B. List of primers used for mutagenesis

	Forward Primer (5'-3')	Reverse Primer (5'-3')
Mutation	GTGAATTCTCTCCTAGaTCcTTGACaTTCCAAGTC ATGTAC	GTACATGACTTGGAAtGTCAAgGAtCTAGGAGAGAATTC AC

bioRxiv preprint doi: https://doi.org/10.1101/2023.11.20.567833; this version posted February 8, 2024. The copyright holder for this preprint (which was not certified by peer review) is the author/funder, who has granted bioRxiv a license to display the preprint in perpetuity. It is made available under aCC-BY 4.0 International license.

C. List of primer sequences for mouse ChIP-qPCR

**Gene**	**Forward Primer (5'-3')**	**Reverse Primer (5'-3')**
FincoR	TCCTAGGTCTTTGACCTTCCAA	TGTGCCCACTGGTGAAATAG
Non-specific region	GTGTACACGCCCAAACTTGA	GTTCACCTCTCTAGCTCACCT

D. List of primer used for RACE

	**Gene specific primer (5'-3')**
5'RACE	GATTACGCCAAGCTT GGGAACTCACGTCTGGAAGCAGAAGCTG
3'RACE	GATTACGCCAAGCTT GGACACTCGGGAATTGACAGTGGGTCAGG
3'RACE nest primer	GATTACGCCAAGCTT GTGGGGGATAGCACAAGCGCCAGGATGC

E. List of primer used for genotyping

**Forward Primer (5'-3')**	**Reverse Primer (5'-3')**
GTGATTTCCTTTCCCACAGC	GTCAGGGAGCTAACGAATGC

bioRxiv preprint doi: https://doi.org/10.1101/2023.11.20.567833; this version posted February 8, 2024. The copyright holder for this preprint (which was not certified by peer review) is the author/funder, who has granted bioRxiv a license to display the preprint in perpetuity. It is made available under aCC-BY 4.0 International license.

Figure 1-figure supplement 1

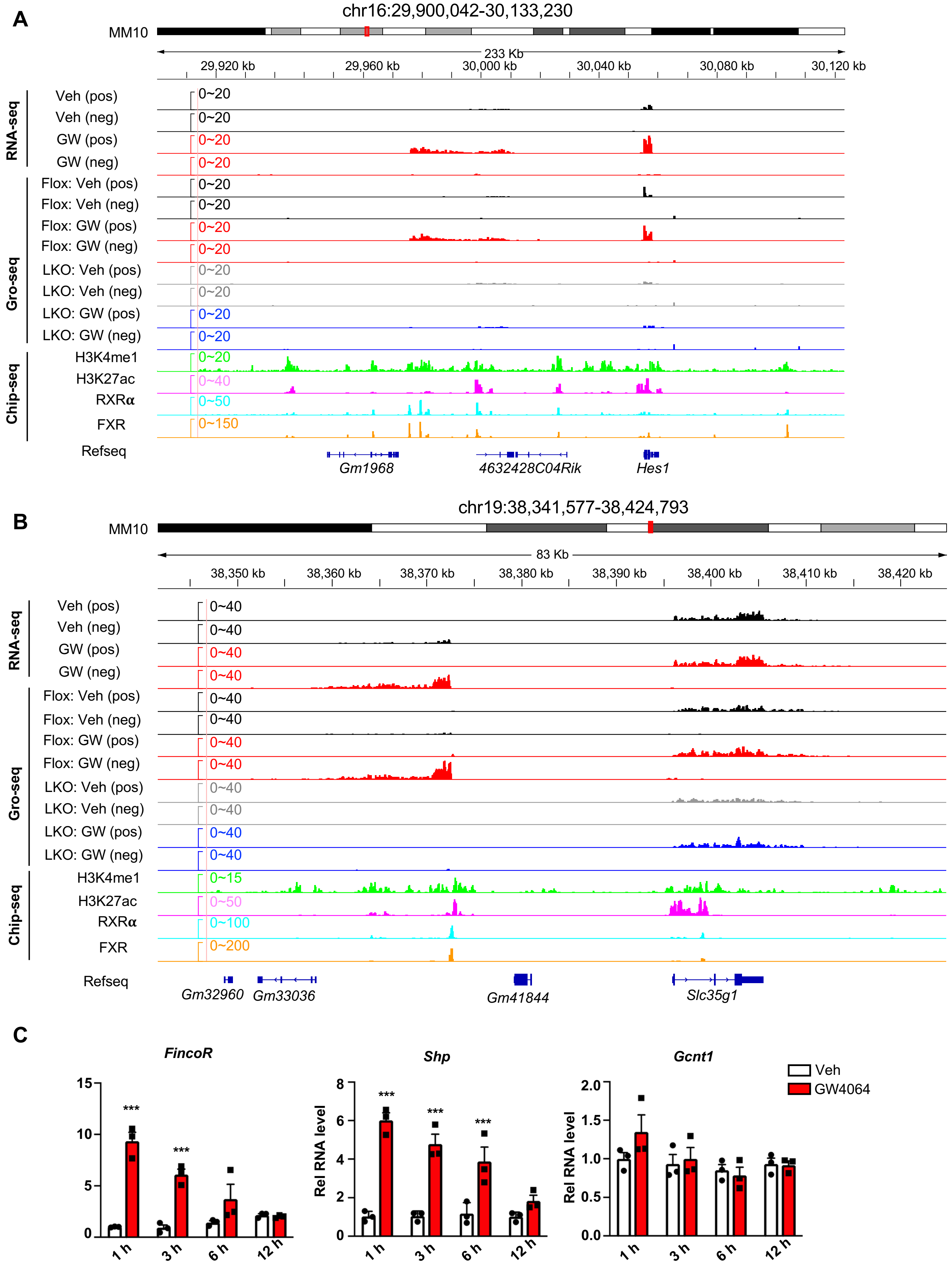

bioRxiv preprint doi: https://doi.org/10.1101/2023.11.20.567833; this version posted February 8, 2024. The copyright holder for this preprint (which was not certified by peer review) is the author/funder, who has granted bioRxiv a license to display the preprint in perpetuity. It is made available under aCC-BY 4.0 International license.

Figure 1–figure supplement 2

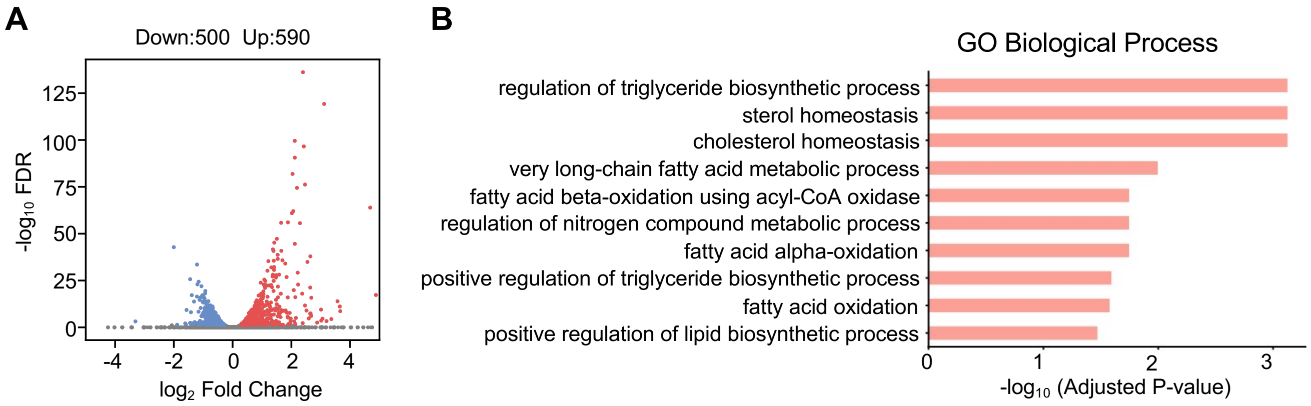

bioRxiv preprint doi: https://doi.org/10.1101/2023.11.20.567833; this version posted February 8, 2024. The copyright holder for this preprint (which was not certified by peer review) is the author/funder, who has granted bioRxiv a license to display the preprint in perpetuity. It is made available under aCC-BY 4.0 International license.

Figure 2-figure supplement 1

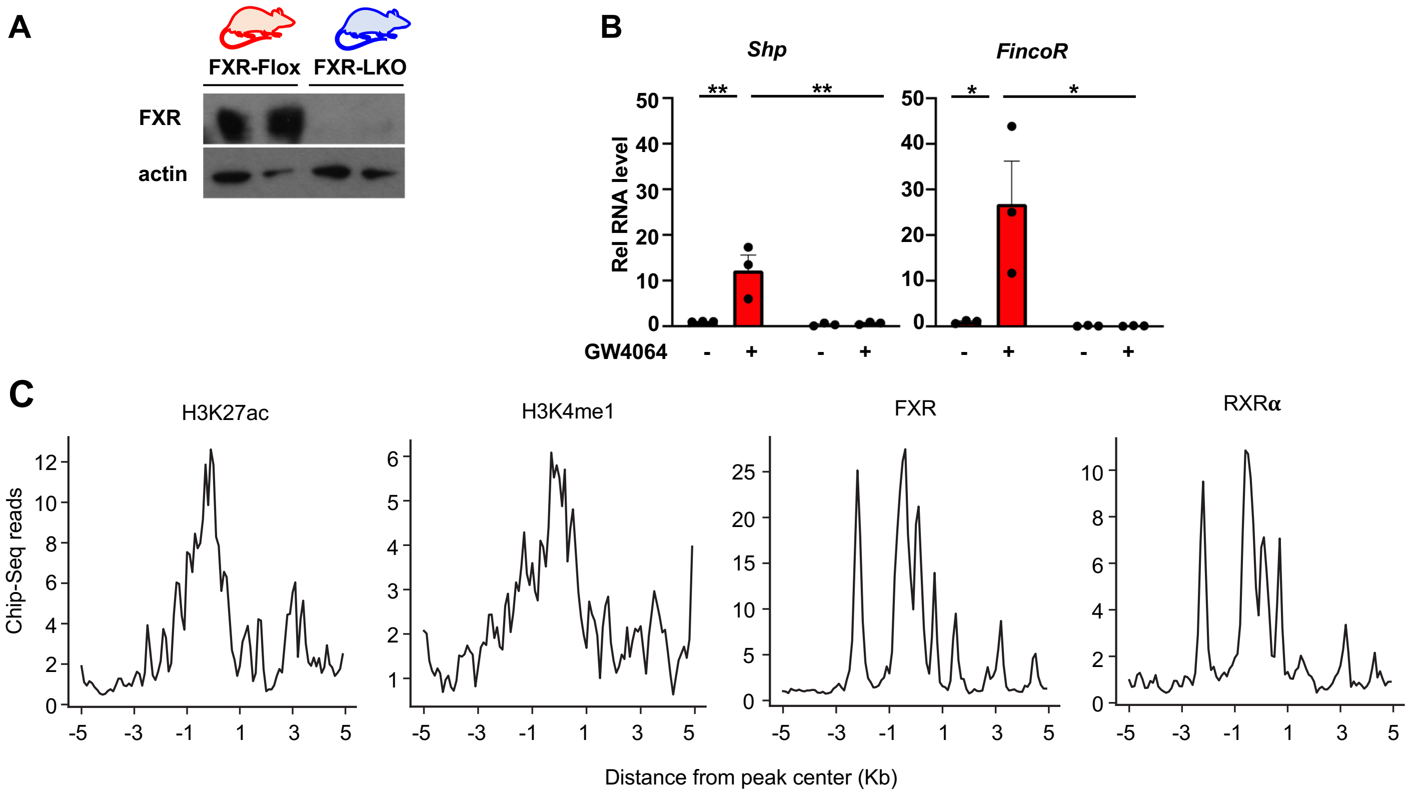

bioRxiv preprint doi: https://doi.org/10.1101/2023.11.20.567833; this version posted February 8, 2024. The copyright holder for this preprint (which was not certified by peer review) is the author/funder, who has granted bioRxiv a license to display the preprint in perpetuity. It is made available under aCC-BY 4.0 International license.

Figure 5-figure supplement 1

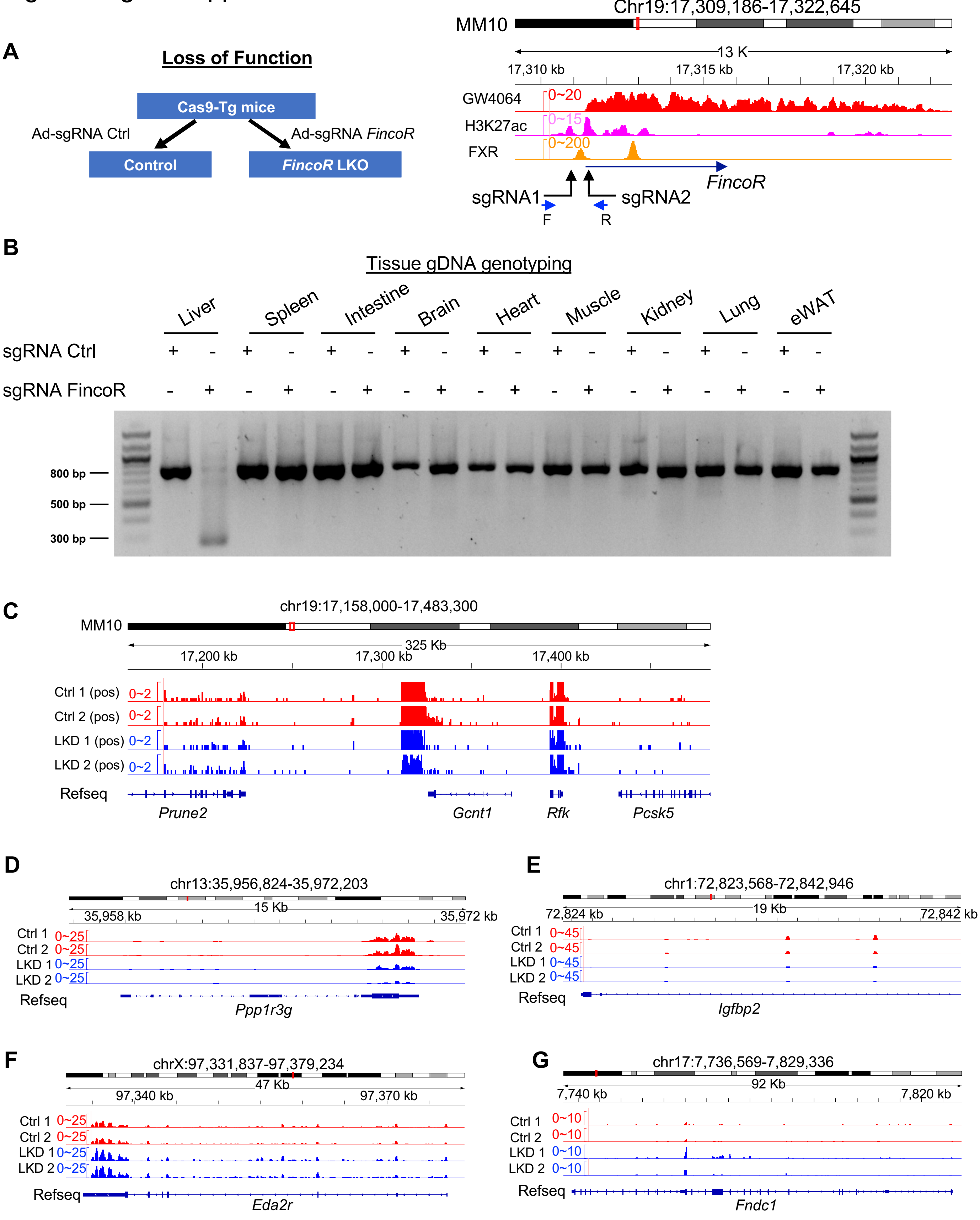

bioRxiv preprint doi: https://doi.org/10.1101/2023.11.20.567833; this version posted February 8, 2024. The copyright holder for this preprint (which was not certified by peer review) is the author/funder, who has granted bioRxiv a license to display the preprint in perpetuity. It is made available under aCC-BY 4.0 International license.

Figure 7-figure supplement 1

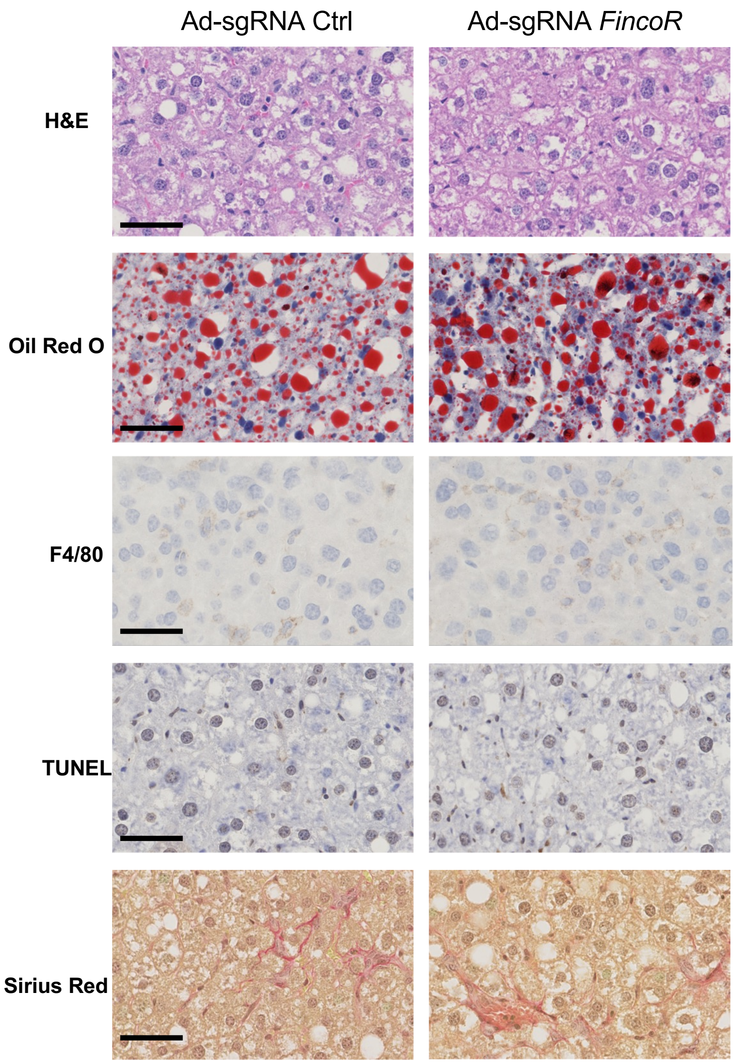

bioRxiv preprint doi: https://doi.org/10.1101/2023.11.20.567833; this version posted February 8, 2024. The copyright holder for this preprint (which was not certified by peer review) is the author/funder, who has granted bioRxiv a license to display the preprint in perpetuity. It is made available under aCC-BY 4.0 International license.

Supplementary Figure S1

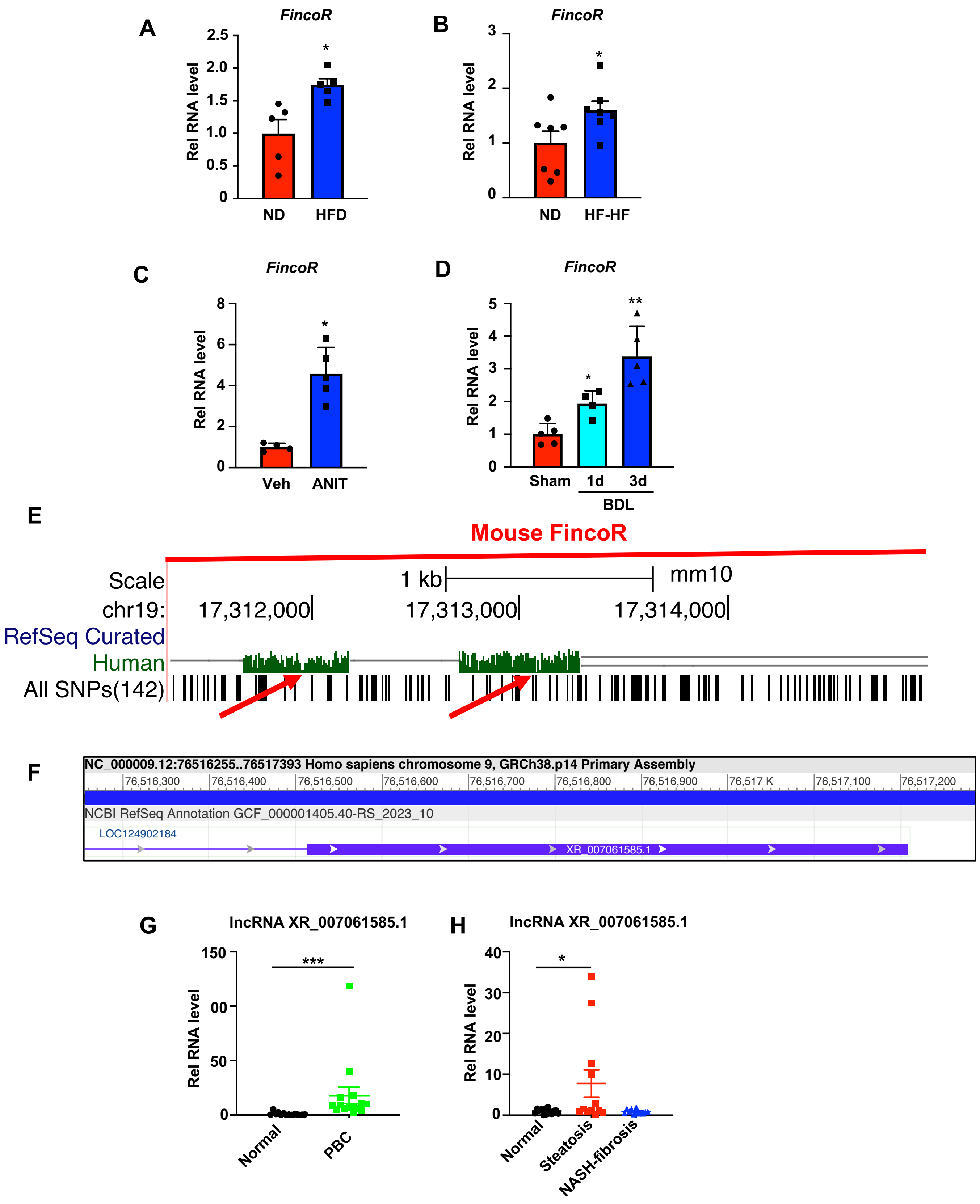

bioRxiv preprint doi: https://doi.org/10.1101/2023.11.20.567833; this version posted February 8, 2024. The copyright holder for this preprint (which was not certified by peer review) is the author/funder, who has granted bioRxiv a license to display the preprint in perpetuity. It is made available under aCC-BY 4.0 International license.

Supplementary Figure S2

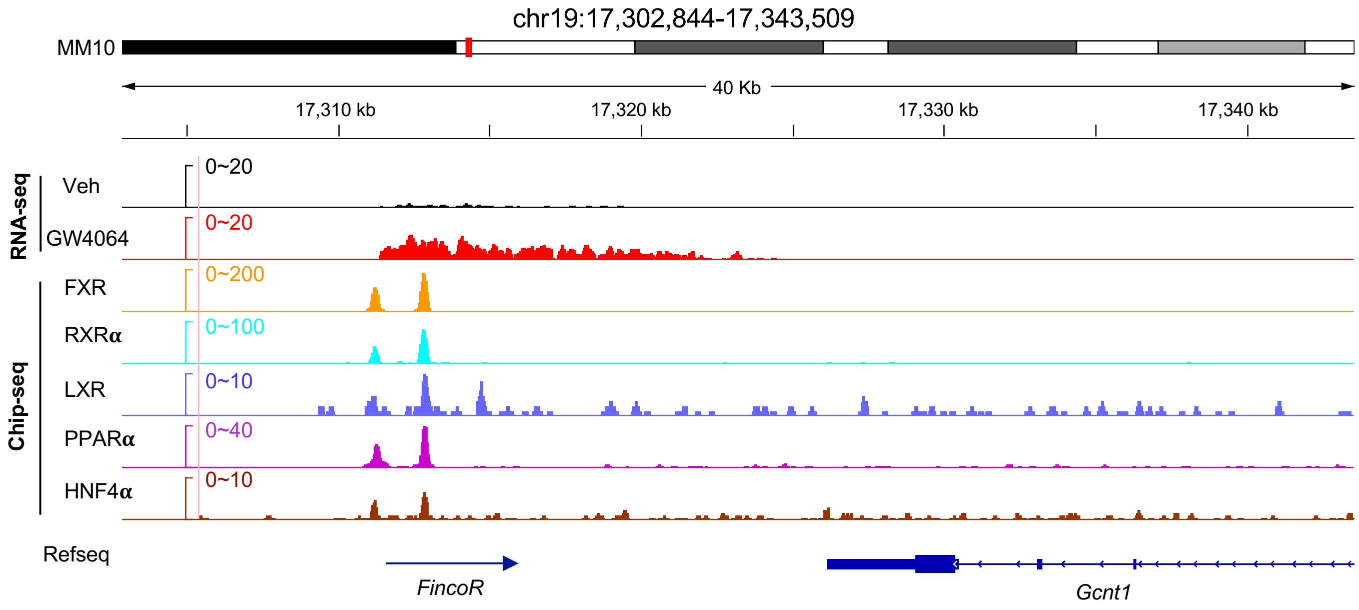

bioRxiv preprint doi: https://doi.org/10.1101/2023.11.20.567833; this version posted February 8, 2024. The copyright holder for this preprint (which was not certified by peer review) is the author/funder, who has granted bioRxiv a license to display the preprint in perpetuity. It is made available under aCC-BY 4.0 International license.

Supplementary Figure S3

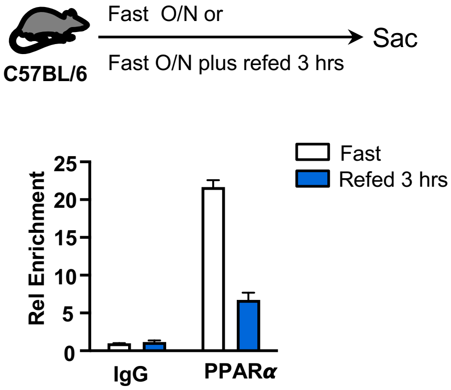

Supplement: Supplement 1 [file NIHPP2023.11.20.567833v2-supplement-1.pdf]
