# Supplementary material for: Hammerhead-type FXR agonists induce an eRNA FincoR that ameliorates nonalcoholic steatohepatitis in mice
Source: bioRxiv. 2023 Nov 21:2023.11.20.567833. Preprint. [Version 1] doi: 10.1101/2023.11.20.567833 (PMC10690184; doi:10.1101/2023.11.20.567833)

bioRxiv preprint doi: https://doi.org/10.1101/2023.11.20.567833; this version posted November 21, 2023. The copyright holder for this preprint (which was not certified by peer review) is the author/funder, who has granted bioRxiv a license to display the preprint in perpetuity. It is made available under aCC-BY 4.0 International license.

Supplementary Figure Legends

Figure 1- Figure Supplement 1. (A, B) Examples of FXR-regulated eRNAs produced near the genes *Hes1* (A) and *Slc35g1* (B) were shown. (C) Time course expression of *FincoR*. C57BL/6 male mice were fasted overnight and *i.p.* injected with vehicle or GW4064 (30 mg/kg) for 1, 3, 6,12 h. Livers were collected at indicated time points (n = 3 mice) and *FincoR*, *Shp* and *Gcnt1* were measured. Data are presented as mean ± SEM. Statistical significance was determined by the two-way ANOVA Sidak's multiple comparisons test with *$p < 0.05$ and ***$p < 0.001$.

Figure 1- Figure Supplement 2. (A) Volcano plot showing the differential expressed genes (DEGs) by GW4064 treatment (DESeq2 FDR<0.05). The numbers refer to the number of genes up- or down-regulated. (B) Bar plot showing the enriched Gene ontology in terms of biological processes for up-regulated genes.

Figure 2- Figure Supplement 1. (A) FXR protein levels in the livers isolated from FXR-Flox and FXR-LKO mice were shown. (B) Validation of hepatic FXR-dependent induction of *FincoR* by qPCR (n = 3 mice). *Shp* gene was used as a control. Data are presented as mean ± SEM. Statistical significance was determined by the two-way ANOVA Tukey's multiple comparisons test with *$p < 0.05$, **$p < 0.01$ and ***$p < 0.001$. (C) Metagene plots showing H3K27ac, H3K4me1, FXR, and RXRα ChIP-Seq profiles centered on up-regulated eRNAs.

Figure 5- Figure Supplement 1. (A) The scheme for the *FincoR* loss of function experiments. (B) The genomic DNAs from liver, spleen, intestine, brain, heart, muscle, kidney, lung and adipose tissue were isolated and PCR was performed using the primers (**Supplementary Table S5E**) to verify tissue-specific knock-out. (C-G) RNA-seq profiles

bioRxiv preprint doi: https://doi.org/10.1101/2023.11.20.567833; this version posted November 21, 2023. The copyright holder for this preprint (which was not certified by peer review) is the author/funder, who has granted bioRxiv a license to display the preprint in perpetuity. It is made available under aCC-BY 4.0 International license.

of expression of hepatic *Prune2* (C), *PPP1r3g* (D), *Igfbp2* (E), *Eda2r* (F) *and Fndc1* (G) were shown (n = 2 mice).

Figure 7- Figure Supplement 1. The effects of FincoR downregulation on NASH pathologies. Male Cas9 mice were fed with NASH diet for 12 weeks. Then these mice were randomly assigned to 2 groups and infected with adenovirus expressing sgRNA for *FincoR* or control. The tissues were collected 2 weeks later. Liver histology analysis was performed. Scale bar (50 μm).

Other Supplementary Figure 1. Hepatic genome browser tracks of FXR, RXRα, LXR, PPARα, and HNF4α binding peaks at *FincoR* locus.

Other Supplementary Figure 2. PPARα occupnacy in the FincoR enhancer region. C57BL/6 male mice were fasted overnight with or without refed for 3 hrs and then sacrificed. ChIP assays were performed in liver samples to detect PPAR**α** occupancy to the FXR binding peak region close to the transcription start site of *FincoR*.

bioRxiv preprint doi: https://doi.org/10.1101/2023.11.20.567833; this version posted November 21, 2023. The copyright holder for this preprint (which was not certified by peer review) is the author/funder, who has granted bioRxiv a license to display the preprint in perpetuity. It is made available under aCC-BY 4.0 International license.

Supplemental Tables

Supplemental Table S1. Sequencing data generated in this study

Name	Total reads	Uniquely mapped reads
WT Liver Veh RNA-Seq rep1	50887391	38072659
WT Liver GW RNA-Seq rep1	54020150	40595856
WT Liver Veh RNA-Seq rep2	51268164	38898913
WT Liver GW RNA-Seq rep2	51899892	39487241
Flox Liver Veh GRO-Seq	62960996	30305910
Flox Liver GW GRO-Seq	59210073	26116795
FXR KO Liver Veh GRO-Seq	34153537	14379417
FXR KO Liver GW GRO-Seq	36430101	15008281
WT Liver RNA-Seq rep1	96262381	81801494
WT Liver RNA-Seq rep2	104979915	88365481
FincoR KD Liver RNA-Seq rep1	91654076	75346167
FincoR KD Liver RNA-Seq rep2	90211420	74745807

Supplemental Table S2 is attached as an Excel file.

Supplemental Table S3. Public ChIP-Seq dataset

target	accession ID
FXR	https://genome.ucsc.edu/cgi-bin/hgCustom?hgsid=1577671033_RPWVA7jbx3rDswKOeo2dazTsXxe0&hgct_table=ct_FxrLiver_2971
H3K27ac	ENCFF001KMI
H3K4me1	ENCFF001KNF
LXR	GSM864669
RXRα	GSM864674
PPARα	GSM864671
HNF4α	GSM2055887

Supplemental Table S4 is attached as an Excel file.

Supplemental Table S5. Primer sequences used in this study

A. List of primer sequences for mouse qRT-PCR

Gene	Forward Primer (5'-3')	Reverse Primer (5'-3')
FincoR	GCAAAGCACCTTCTAGCACA	GTCAGGGAGCTAACGAATGC
Gcnt1	GCATCGCATCCTGCTTTGATA	GGTCTGCCTTAACCCGACTC
Shp	TCTGCAGGTCGTCCGACTAT	CAGGCAGTGGCTGTGAGAT
Cyp7a1	GGGATTGCTGTGGTAGTGAGC	GGTATGGAATCAACCCGTTGTC

bioRxiv preprint doi: https://doi.org/10.1101/2023.11.20.567833; this version posted November 21, 2023. The copyright holder for this preprint (which was not certified by peer review) is the author/funder, who has granted bioRxiv a license to display the preprint in perpetuity. It is made available under aCC-BY 4.0 International license.

Cyp8b1	CCTCTGGACAAGGGTTTTGTG	GCACCGTGAAGACATCCCC
Srebp1c	GCAGCCACCATCTAGCCTG	CAGCAGTGAGTCTGCCTTGAT
Lpin	CATGCTTCGGAAAGTCCTTCA	GGTTATTCTTTGGCGTCAACCT
Scd1	TTCTTGCGATACACTCTGGTGC	CGGGATTGAATGTTCTTGTCGT
Ifng	GCG TCA TTG AAT CAC ACC TG	GAC CTG TGG GTT GTT GAC CT
Ccl3	TTCTCTGTACCATGACACTCTGC	CGTGGAATCTTCCGGCTGTAG
Ccl2	TTAAAAACCTGGATCGGAACCAA	GCATTAGCTTCAGATTTACGGGT
Ccr2	AAGAGGGCATTGGATTCACCACA	GCCGTGGATGAACTGAGGTAACA
Lcn2	GCAGGTGGTACGTTGTGGG	CTCTTGTAGCTCATAGATGGTGC
Col1a1	ATCGGTCATGCTCTCTCCAAACCA	ACTGCAACATGGAGACAGGTCAGA
Col1a2	CCTTTGTCAGAATACTGAGCAGC	GTAACTTCGTGCCTAGCAACA
Acta2	TCGGATACTTCAGCGTCAGGA	GTCCCAGACATCAGGGAGTAA
Eda2r	CACACTGCATAGTCTGCCCTC	GCCTTCTGGACCCGATTGA
Fndc1	GGGAGACATGGCAAACCTGT	TGGTAGGAGAGTATGTGGTGG
Ctsb	TCCTTGATCCTTCTTTCTTGCC	ACAGTGCCACACAGCTTCTTC
Ctss	GAAGTACGGCGTCTCATCTGG	CATGCCCACTTGGTAGGTATG
Bcl2	GTCGCTACCGTCGTGACTTC	CAGACATGCACCTACCCAGC
36b4	CCCTGAAGTGCTCGACATCA	TGCGGACACCCTCCAGAA

B. List of primers used for mutagenesis

	Forward Primer (5'-3')	Reverse Primer (5'-3')
Mutation	GTGAATTCTCTCCTAGaTCcTTGACaTTCCAAGTC ATGTAC	GTACATGACTTGGAAtGTCAAgGAtCTAGGAGAGAATTC AC

C. List of primer sequences for mouse ChIP-qPCR

**Gene**	**Forward Primer (5'-3')**	**Reverse Primer (5'-3')**
FincoR	TCCTAGGTCTTTGACCTTCCAA	TGTGCCCACTGGTGAAATAG
Non-specific region	GTGTACACGCCCAAACTTGA	GTTCACCTCTCTAGCTCACCT

D. List of primer used for RACE

	**Gene specific primer (5'-3')**
5'RACE	GATTACGCCAAGCTT GGGAACTCACGTCTGGAAGCAGAAGCTG
3'RACE	GATTACGCCAAGCTT GGACACTCGGGAATTGACAGTGGGTCAGG
3'RACE nest primer	GATTACGCCAAGCTT GTGGGGGATAGCACAAGCGCCAGGATGC

bioRxiv preprint doi: https://doi.org/10.1101/2023.11.20.567833; this version posted November 21, 2023. The copyright holder for this preprint (which was not certified by peer review) is the author/funder, who has granted bioRxiv a license to display the preprint in perpetuity. It is made available under aCC-BY 4.0 International license.

E. List of primer used for genotyping

Forward Primer (5'-3')	Reverse Primer (5'-3')
GTGATTTCCTTTCCCACAGC	GTCAGGGAGCTAACGAATGC

bioRxiv preprint doi: https://doi.org/10.1101/2023.11.20.567888; this version posted November 21, 2023. The copyright holder for this prep
(which was not certified by peer review) is the author/funder, who has granted bioRxiv a license to display the preprint in perpetuity. It is ma
available under aCC-BY 4.0 International license.

Figure 1-Figure Supplement 1

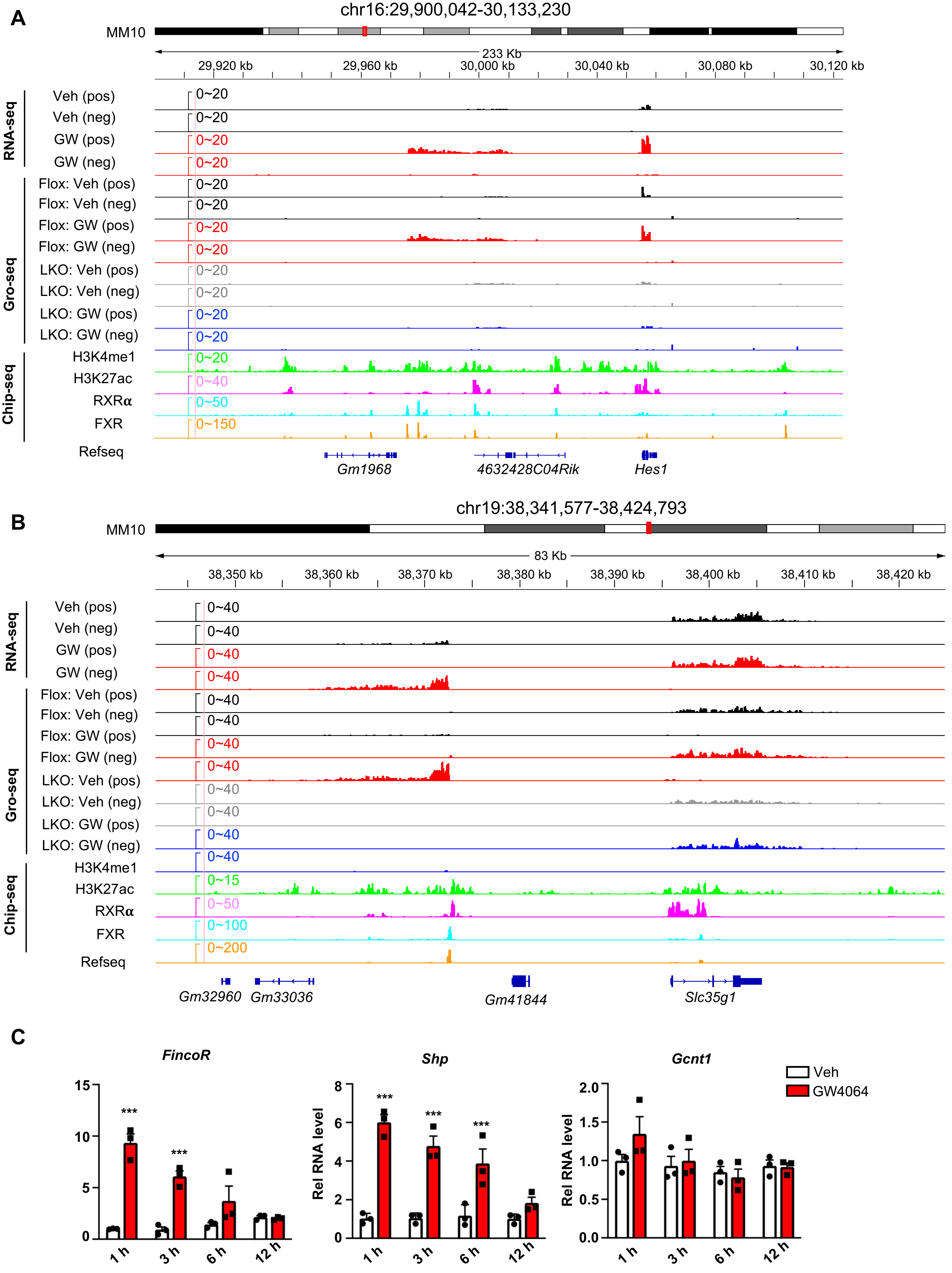

(which was not certified by peer review) is the author/funder, who has granted bioRxiv a license to display the preprint in perpetuity. It is m
available under aCC-BY 4.0 International license.

Figure 1–Figure Supplement 2

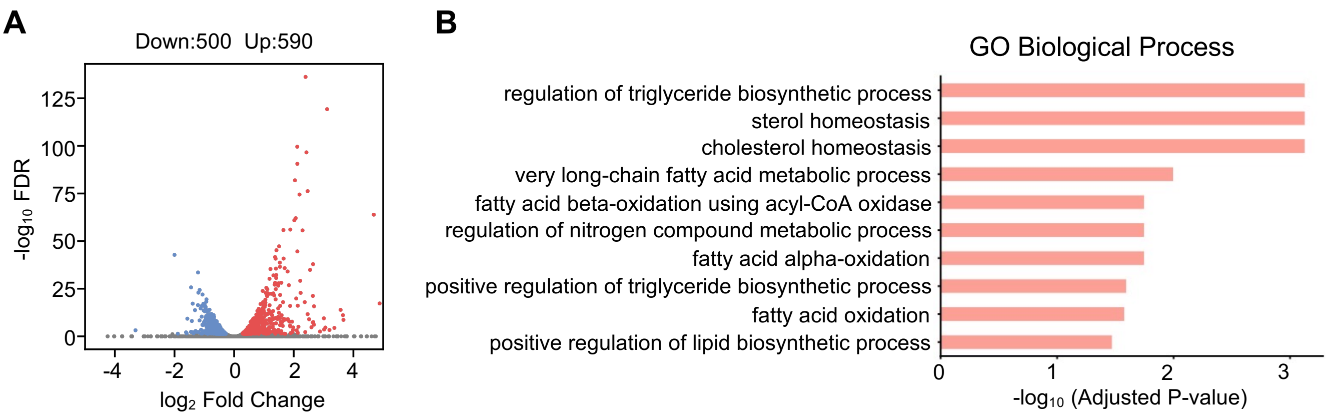

bioRxiv preprint doi: https://doi.org/10.1101/2023.11.20.567968; this version posted November 22, 2023. The copyright holder for this preprint (which was not certified by peer review) is the author/funder, who has granted bioRxiv a license to display the preprint in perpetuity. It is made available under aCC-BY 4.0 International license.

Figure 2-Figure Supplement 1

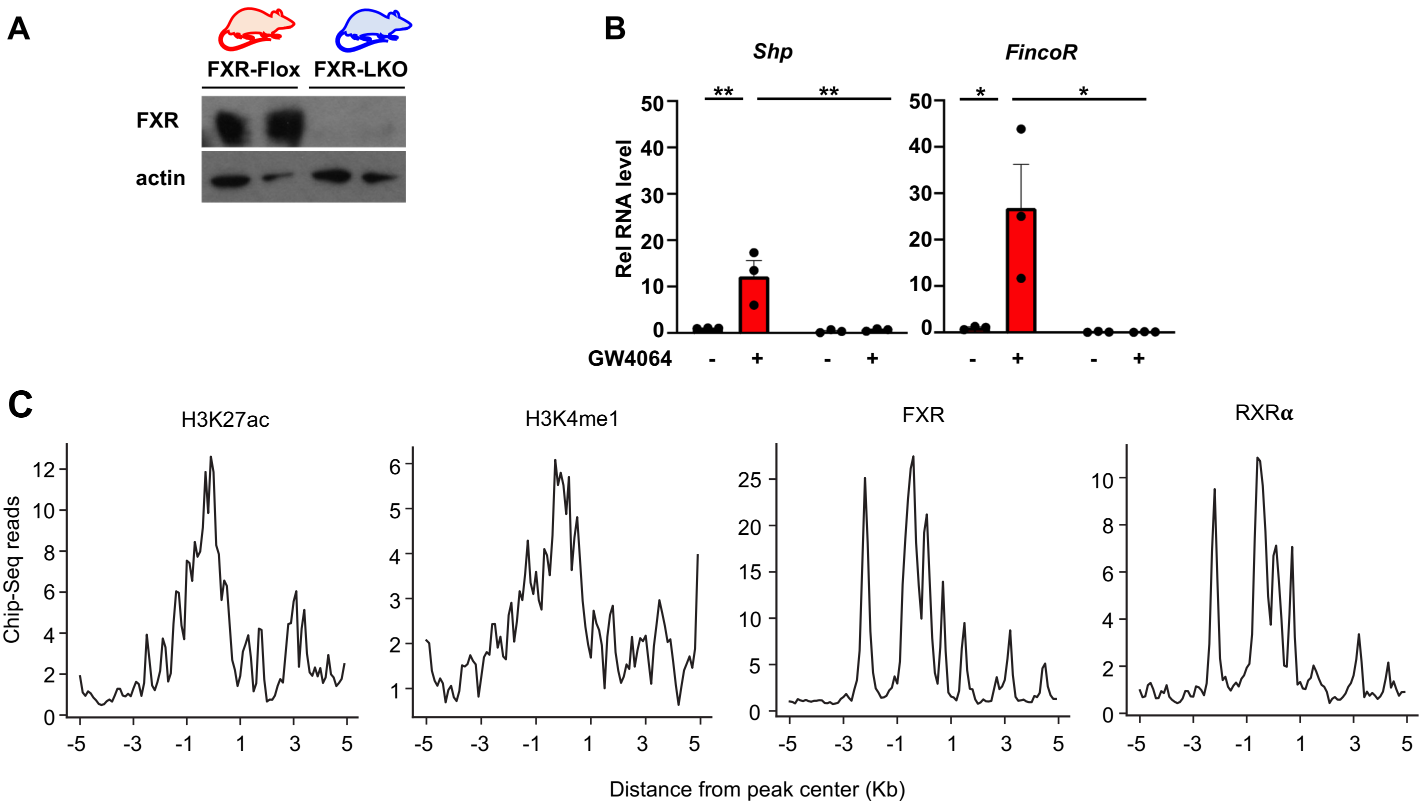

bioRxiv preprint doi: https://doi.org/10.1101/2023.11.20.[illegible]; this version posted November 21, 2023. The copyright holder for this prep[illegible]
(which was not certified by peer review) is the author/funder, who has granted bioRxiv a license to display the preprint in perpetuity. It is ma[illegible]
available under aCC-BY 4.0 International license.

Figure 5-Figure Supplement 1

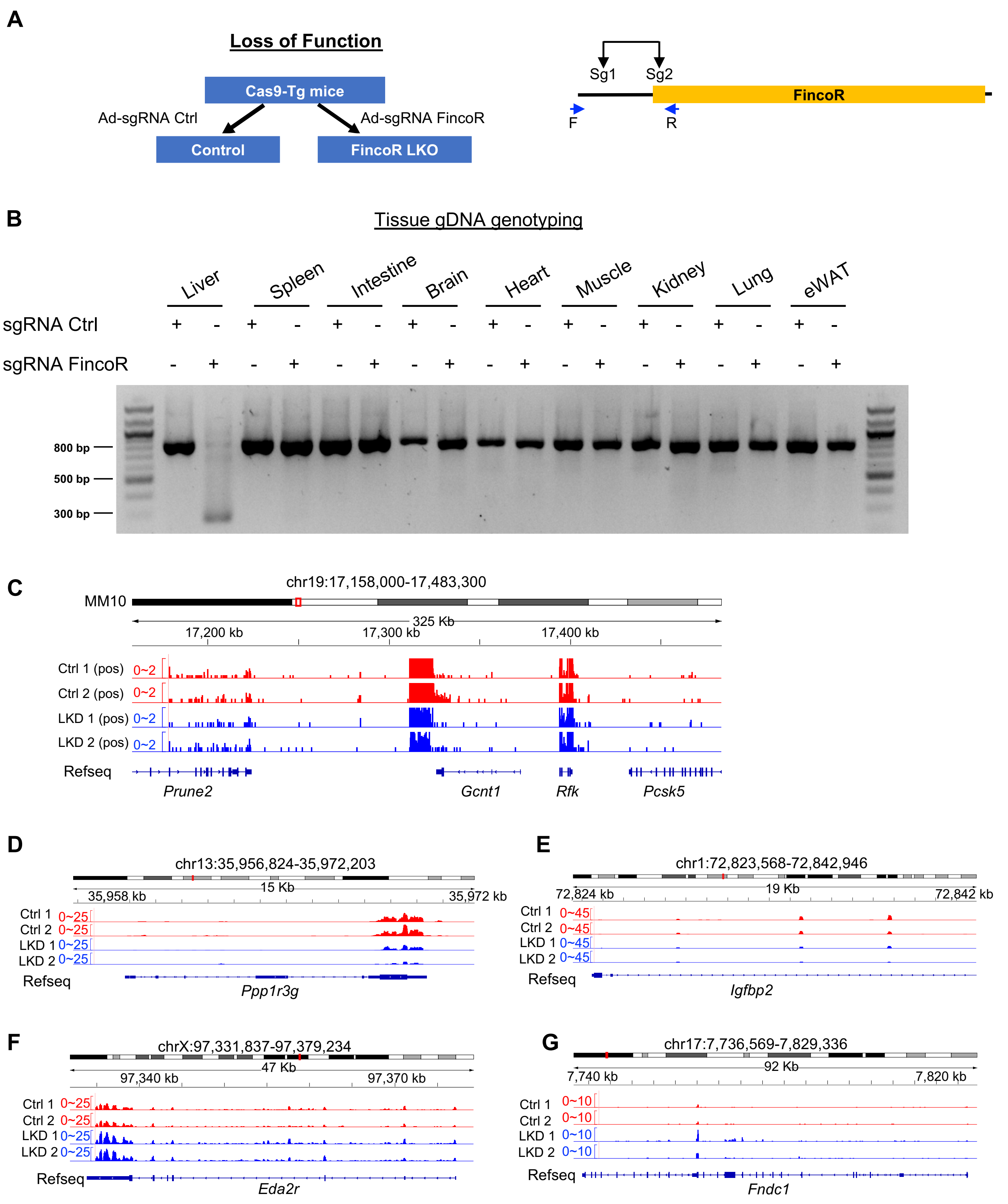

bioRxiv preprint doi: https://doi.org/10.1101/2023.11.20.567888; this version posted November 22, 2023. The copyright holder for this preprint (which was not certified by peer review) is the author/funder, who has granted bioRxiv a license to display the preprint in perpetuity. It is made available under aCC-BY 4.0 International license.

Figure 7-Figure Supplement 1

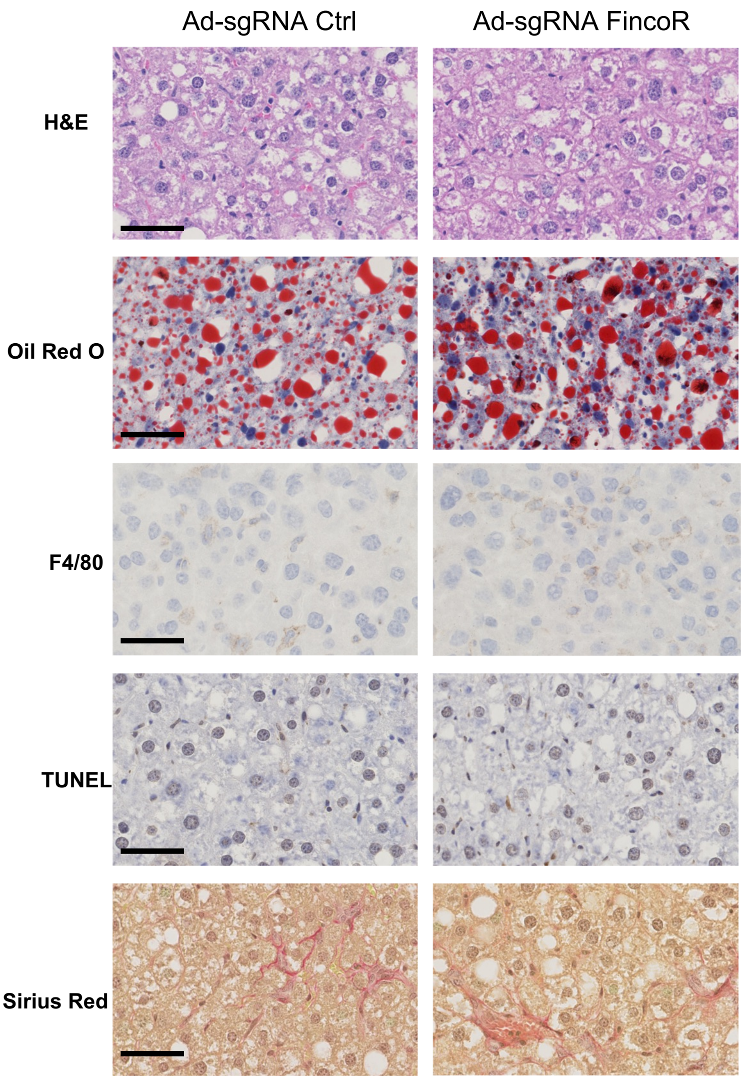

[illegible]
(which was not certified by peer review) is the author/funder, who has granted bioRxiv a license to display the preprint in perpetuity. It is m
available under aCC-BY 4.0 International license.

Other Figure Supplement 1

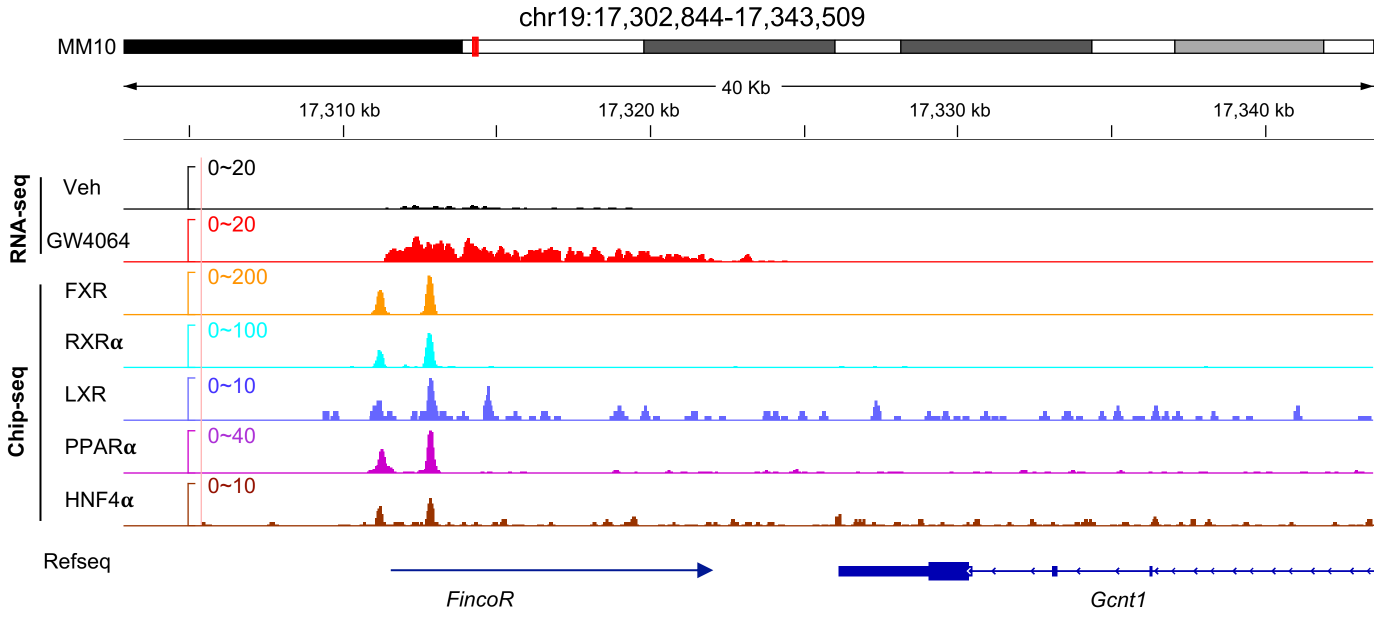

[illegible]
(which was not certified by peer review) is the author/funder, who has granted bioRxiv a license to display the preprint in perpetuity. It is m
available under aCC-BY 4.0 International license.

Other Figure Supplement 2

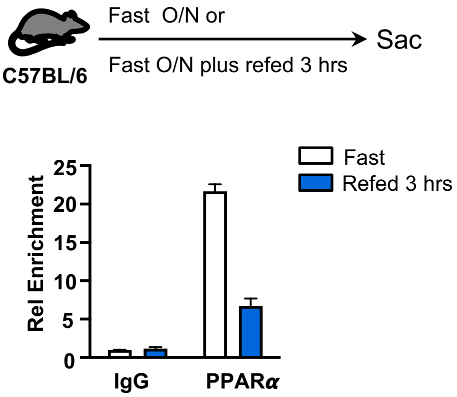

Supplement: Supplementary file 1 [file NIHPP2023.11.20.567833V1-supplement-1.pdf]
